# Nanotoxicological Assessment of Green-Synthesized Silver Nanoparticles from Brazilian Cerrado Plant in a Murine Model

**DOI:** 10.3390/ph18070993

**Published:** 2025-07-02

**Authors:** Cínthia Caetano Bonatto, Ivy Garcez Reis, Dalila Juliana Silva Ribeiro, Raquel das Neves Almeida, Rafael Corrêa, Livia Pimentel Sant’Ana Dourado, Gabriel Pasquarelli-do-Nascimento, Kelly Grace Magalhães, Luciano Paulino Silva

**Affiliations:** 1 Postgraduate Program in Animal Biology, University of Brasilia (UnB), Brasília 70910-900, DF, Brazil; 2 Laboratory of Nanobiotechnology (LNANO), Embrapa Genetic Resources and Biotechnology, Parque Estacao Biologica, Final W5 Norte, Brasília 70770-917, DF, Brazil; 3 Institute of Biological Sciences, University of Brasilia (UnB), Brasília 70910-900, DF, Brazil; 4 Laboratory of Immunology and Inflammation, Department of Cell Biology, University of Brasilia (UnB), Brasília 70910-900, DF, Brazil; 5 Institute of Innate Immunity, Biophysical Imaging, Medical Faculty, University of Bonn, Venusberg-Campus 1, 53127 Bonn, Germany; 6 Neuroimmune Interactions Laboratory, Department of Neurology, UMass Chan Medical School, Worcester, MA 01655, USA

**Keywords:** AgNPs, *Caryocar brasiliense*, nanosafety, Cerrado, green synthesis, hematological analysis, biochemical analysis, histological analysis

## Abstract

**Background/Objectives:** In recent years, silver nanoparticles (AgNPs) have garnered significant attention due to their potent antimicrobial properties, which hold promise for various applications. However, concerns about their potential toxicity have also emerged, particularly regarding their impact on human and animal health. This study investigates the acute toxicological effects of AgNPs synthesized using a green route with an aqueous extract of a native Cerrado plant (AgNPs-Cb) in mice. **Methods**: The AgNPs-Cb were intravenously administered at a concentration of 64 µM, and the mice were euthanized after 24 h for the collection of blood and organ samples (liver, spleen, kidneys, and lungs) for hematological, biochemical, and histological analyses. **Results:** Hematological analysis, including complete blood count (CBC) and differential leukocyte count, showed no statistically significant alterations in the groups treated with AgNPs-Cb, Cb extract, and Ag^+^, compared with the control group (*p* < 0.05). Notably, only the Ag^+^ group exhibited a significant increase in red blood cell count and hematocrit levels, suggesting that the nanoformulation of silver might mitigate the hematological impact seen with free silver ions. Biochemical analyses of liver and kidney function markers also revealed no significant differences across the treatment groups. **Conclusions:** These findings indicate that AgNPs-Cb may offer a safer alternative for antimicrobial applications, reducing the risk of acute toxicity in mammals while maintaining efficacy against pathogens. Further studies are needed to explore the underlying mechanisms and long-term effects of AgNPs-Cb exposure.

## 1. Introduction

In the last decade, silver nanoparticles (AgNPs) have attracted great interest in academia and industry due to their unique physicochemical and biological characteristics, which provide a range of possibilities for use in various applications. For example, AgNPs can be incorporated into optical fibers, superconducting materials, cosmetic products, food industries, and electronic components, among others [1]. In addition, they are used extensively in the biomedical area, where they are added to dressings, creams, antiseptic sprays, and fabrics due to their broad-spectrum biocidal effect against microorganisms, particularly bacteria [1].

With this arsenal of potential uses, human, animal, plant, and environmental exposures to AgNPs have also risen, especially since they have been reported to potentially accumulate and persist for extended periods in biological and environmental systems [1,2,3]. AgNPs nanotoxicity is substantially influenced by a range of physicochemical properties, including particle size, shape, surface charge, aggregation or agglomeration state, crystallinity, surface coating, functionalization, composition, among others [3]. These characteristics directly affect their biological interactions, cellular uptake, biodistribution, and, ultimately, the toxicity profile of AgNPs within biological systems [4,5]. However, numerous studies have yielded varying results regarding their toxicity, which may be related to the diversity of synthesis routes used to obtain AgNPs, whether physical, chemical, or biological (green) [6].

Green synthesis refers to environmentally friendly routes for nanomaterial production that employ relatively nontoxic, biodegradable, and cost-effective chemicals, with biological organisms or their components (organs, tissues, cells, or biomolecular metabolites) as the primary agents of reduction and stabilization of AgNPs. Biological resources, including plants, animal derivatives, algae, fungi, bacteria, and a variety of agricultural by-products, have shown potential for the green synthesis of AgNPs via redox processes in aqueous media. The green synthesis of AgNPs using plant extracts or whole plants has been increasingly reported in the literature [7,8,9,10].

*Caryocar brasiliense* Camb., a native species of the Brazilian savannah (Cerrado), holds substantial economic importance, partly due to the unique flavor of its edible pulp, widely used in culinary applications [11]. Traditionally, *C. brasiliense* has been used primarily for its fruit or seed, while the vast majority of its biomass, such as leaves, remains an underutilized by-product [12]. The leaves contain diverse biomolecules, including flavonoids, saponins, xanthones, catechins, steroids, phenols, and polyphenols, representing a promising source for the eco-friendly synthesis of AgNPs [13,14].

In this context, understanding the toxicological effects of AgNPs synthesized from biological resources in a murine model helps elucidate how these eco-friendly nanoparticles interact with living organisms, offering valuable insights into their biocompatibility, biodistribution, and potential adverse toxic effects. Although there has been significant progress in the research on metal nanoparticles (MNPs), particularly silver-based nanoparticles, gaps remain in understanding how their physicochemical properties affect nanotoxicity. This lack of understanding limits the ability to utilize their unique properties in the biomedical field. Addressing these challenges is essential for advancing theoretical frameworks and for improving practical applications. This study aims to address the existing limitations by examining the toxicological effects of AgNPs synthesized from biological resources in a murine model. The goal is to elucidate how these eco-friendly nanoparticles interact with living organisms, providing valuable insights into their biocompatibility, biodistribution, and potential adverse toxic effects.

## 2. Results and Discussion

### 2.1. Silver Nanoparticles Characterization

**Figure 1:**
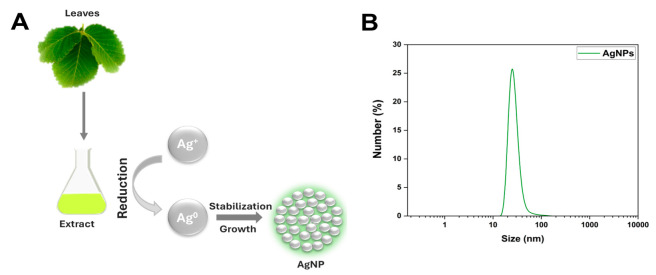
Schematic representation of green-synthesized silver nanoparticles from leaves of a Brazilian Cerrado plant (**A**); distribution curve of hydrodynamic diameters of AgNPs-Cb, presented as a number (%) obtained through dynamic light scattering (**B**).

In green synthesis approaches, biological components (e.g., primary and secondary metabolites) function as agents to promote the reduction of metal ions (Ag^+^) to neutral metals (Ag^0^), resulting in the formation of AgNPs (Figure 1A). In addition, biomolecules present in the reaction medium can also act as stabilizing and surface-coating agents for AgNPs (Figure 1A), preventing or at least minimizing the agglomeration, aggregation, or a combination of both processes during and at the end of the synthesis process [10,15,16].

Optical properties, either as the direct color change in the colloidal suspensions detected visually or by absorption spectrophotometry, are among the approaches used to evaluate the success of AgNP formation [17]. A reaction between Cb and AgNO_3_ resulted in a color change from translucent to reddish-brown, indicating the formation of AgNPs-Cb. The maximum absorbance band observed at 410 nm indicated AgNP formation and corresponded to the surface plasmon resonance (SPR). The nanometer size and moderate polydispersity of the AgNPs-Cb were verified using DLS analysis, revealing an average hydrodynamic diameter of 38.37 ± 4.61 nm, unimodal distribution in hydrodynamic diameter class intervals at a value of 23 nm (Figure 1B), and a PdI of 0.399 ± 0.007. Additionally, the AgNPs-Cb exhibited a zeta potential of −35.9 ± 1.0 mV, indicating moderate colloidal stability.

**Figure 2:**
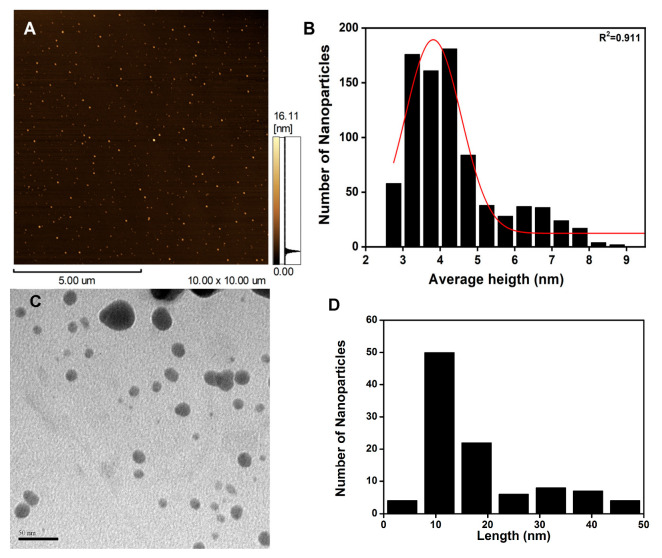
Atomic force microscopy top-view image (**A**); histogram obtained through the AFM height of AgNPs-Cb, with the red line indicating the Gauss-fitted distribution curve (**B**); representing the class distribution profile, transmission electron microscopy image showing quasi-spherically shaped AgNPs-Cb (**C**); histogram of the TEM length (dry diameter) of AgNPs-Cb (**D**).

Numerous studies have demonstrated that the refractive plasmonic scattering (RPS) effect is closely linked to the shape and size of MNPs, including AgNPs [18,19,20]. In this study, atomic force microscopy (AFM) and transmission electron microscopy (TEM) analyses revealed that the AgNPs-Cb exhibited a spherical shape (Figure 2). Due to the low particle count in TEM images, statistical fitting was unfeasible. Thus, the size-distribution quantitative analysis (average) was exclusively performed using AFM (Figure 2B). Furthermore, the AgNPs showed an average height of 4.39 ± 0.05 nm, approximately five times smaller than the sizes determined by DLS. It is important to note that the larger size indicated by DLS is attributed to the coating and solvation layer surrounding the particle surface, as these measurements were taken in a liquid medium where such layers contribute to the hydrodynamic diameter. The smaller AFM height (4.39 nm) versus hydrodynamic diameter (38 nm) reflects the solvation layer and phytochemical coating around AgNPs-Cb in suspension, consistent with spherical morphology. To further understand the interactions of AgNPs-Cb with biological systems, their hemolytic activity was evaluated to assess potential toxicity to blood components.

### 2.2. Hemolytic Activity

Blood is a complex connective tissue composed of diverse cell types and non-cellular components, each playing specific and essential roles in maintaining physiological homeostasis. Among the cells, red blood cells (RBCs) are the most abundant. Recent studies have examined various interactions of AgNPs with blood components, including hemolysis (red blood cell lysis), protein corona formation, nanoparticle aggregation and agglomeration, platelet interactions, and effects on coagulation cascades [21,22]. The hemolytic activity of AgNPs-Cb was evaluated to investigate their potential toxicity to circulating cells in mice blood.

**Figure 3:**
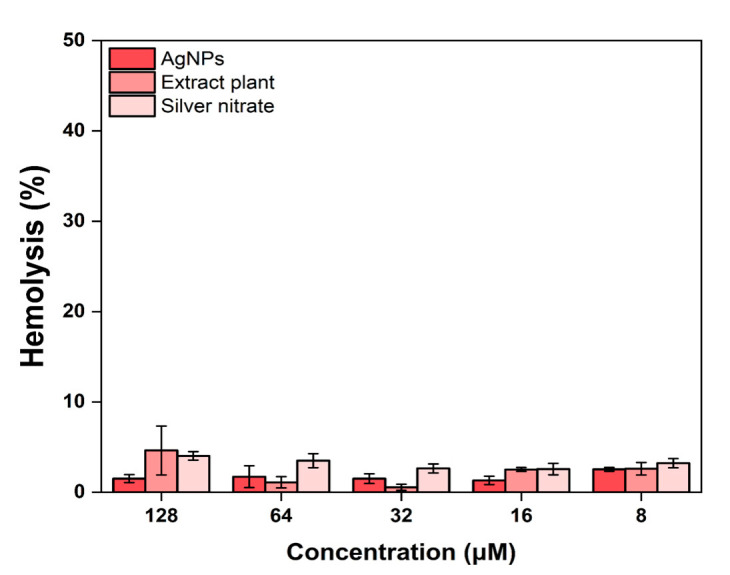
In vitro assessment of the hemolysis percentages using murine RBCs exposed to AgNPs-Cb, Cb, and free Ag^+^. Values are presented as the mean ± standard error of the mean of triplicates.

AgNPs-Cb, Cb, and free Ag^+^ were diluted to various concentrations and incubated with murine blood (strain C57Bl/6) to assess potential hemolysis. No dose-dependent relationship was observed for any of the tested samples, with the AgNPs-Cb showing negligible hemolytic effects, as 98.5% of cells remained intact even at the highest tested concentration (128 μM) (Figure 3), indicating a low hemolytic profile. These results contrast with Huang et al. who reported dose-dependent hemolysis for chemically synthesized 20 nm AgNPs [21]. Differences in hemolytic activity may be attributed to the distinct physicochemical properties of AgNPs synthesized via green methods in this study, as opposed to conventional chemical synthesis. Building on these findings, an in vivo toxicity assessment explored the broader systemic impacts of AgNPs-Cb exposure.

### 2.3. Toxicity Assessment of AgNPs in Mice in Vivo

Over the last decade, AgNPs have garnered significant interest within academia and industry due to their potent antimicrobial properties, which offer diverse applications. However, concerns regarding their potential toxicity have grown due to increasing exposure among humans and other vertebrates, prompting ongoing debates about their safety and health impact [23,24].

To investigate the acute toxicological effects of AgNPs, an in vivo study was conducted using mice. AgNPs-Cb, Cb, and AgNO_3_ were administered intravenously via the caudal vein at a concentration of 64 μM, assuming an approximate blood volume of 2.5 mL per animal. After 24 h, the animals were euthanized, and blood samples, as well as organs (liver, spleen, kidney, and lung), were collected for hematological, biochemical, and histological analyses.

Due to its specialized fluidness as a connective tissue, blood, as well as its hematological parameters, such as complete blood count and leukogram, are critical indicators in nanotoxicology studies. When AgNPs are administered intravenously, they first interact with blood components. These initial interactions may trigger responses from these exogenous agents, potentially leading to acute inflammatory reactions [25,26,27].

In this study, hematological parameters—specifically blood counts (red series) and leukograms (white series)—revealed no statistically significant changes in groups receiving intravenous AgNPs-Cb, Cb, or Ag^+^ compared with the control or reference standards for most evaluated parameters (*p* < 0.05; Table 1 and Table 2). Significant increases in red blood cell count and hematocrit were only observed in the Ag^+^-treated group compared with the control group (Table 1). These findings contrast with those reported in the study by Park in 2013, which observed decreases in red blood cell counts, hematocrit, and hemoglobin, as well as increases in platelet count and volume after oral administration in groups that received Ag^+^ and AgNPs, respectively [28].

Although this result lacks a direct correlation with similar findings in the literature, increased red blood cell count (polycythemia) and hematocrit levels are typically associated with heightened demands for oxygen uptake, transport, or tissue distribution. Given the short 24 h timeframe, however, there may have been insufficient time for these homeostatic adjustments, which usually involve hormonal (e.g., erythropoietin) and bone marrow responses. Further studies are warranted to elucidate the mechanisms underlying these findings. Additionally, since AgNPs did not exhibit the same effects as free silver ions, it is plausible that nanostructuring mitigated these impacts. Next, biochemical markers were analyzed to further investigate potential organ-specific toxicity, focusing on impacts on the liver, kidney, and other systems.

In addition to hematological evaluation, biochemical marker measurement has been widely used to monitor and diagnose potential organ damage [25,28,29]. This study incorporated a comprehensive analysis of several biochemical markers, including gamma-glutamyl transpeptidase (GGT), aspartate aminotransferase (AST), alanine aminotransferase (ALT), total bilirubin (TBIL), direct bilirubin (DBIL), indirect bilirubin (IBIL), blood glucose, total cholesterol and its fractions (low-density lipoproteins (LDLs) and high-density lipoproteins (HDLs)), and triglycerides. The objective was to assess potential damage to critical organs, such as the liver, spleen, and kidney (Table 3 and Table 4).

The results of the biochemical parameter analysis revealed no statistically significant changes in the groups treated with intravenous administrations of AgNPs-Cb, Cb, and Ag^+^ compared with the control group or reference values (Table 3 and Table 4; *p* < 0.05). These findings align with previous research by Xue et al., who demonstrated that intravenous administration of AgNPs at low concentrations (7.5 and 30 mg/kg) in mice did not induce significant alterations in biochemical parameters, including creatinine, AST, lactate dehydrogenase, total cholesterol, and total protein [30].

This consistency in outcomes underscores the potential safety profile of AgNPs-Cb at the tested concentrations. However, to complement the biochemical evaluation and provide a more in-depth understanding of any subclinical effects, histological analysis was employed to investigate tissue-level alterations in the liver, spleen, kidneys, and lungs. This transition to histological analysis allows for a direct examination of cellular and structural integrity, offering a detailed assessment beyond biochemical markers.

### 2.4. Histological Analysis

Histological analyses of organs and tissues are widely used in in vivo studies with AgNPs to assess the structural aspects related to their potential toxicities [23,30,31,32,33]. AgNPs have been reported to accumulate primarily in the spleen, liver, lungs, and kidneys [30,34]. Therefore, to complement the findings from hematological and biochemical evaluations and investigate potential alterations, the organs (liver, spleen, lungs, and kidneys) of the animals were collected and processed for histological examination. The morphological characteristics of the organs were observed by light microscopy using sections stained with the H&E method, where the organs from untreated control animals served as reference points for comparison with those from animals treated with AgNPs-Cb, Cb, or Ag^+^.

**Figure 4:**
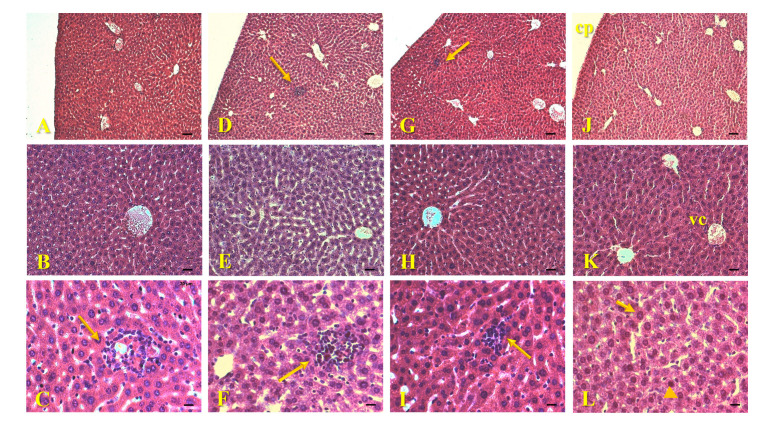
Histological images of mice liver (C57Bl/6) 24 h after intravenous administration of 64 μM AgNPs-Cb (**A**–**C**), Cb (**D**–**F**), AgNO_3_ (**G**–**I**), and the untreated control group (**J**–**L**). The sections show intact capsules (cp) and preserved parenchymal regions (**A**,**D**,**G**,**J**), containing centrilobular veins (vc), hepatocytes (small arrow), and sinusoidal capillaries (arrowhead) (**B**,**C**,**E**,**F**,**H**,**I**,**K**,**L**), and the presence of small inflammatory infiltrates (large arrows) in the AgNPs-Cb (**C**), Cb (**F**), and AgNO_3_ (**I**) groups. Staining: H&E. Scale bars = 50 μm (**A**,**D**,**G**,**J**); 25 μm (**B**,**E**,**H**,**K**); and 12.5 μm (**C**,**F**,**I**,**L**). Sections thickness = 5 μm.

Histological sections of the livers of animals exposed to AgNPs-Cb, Cb, or Ag^+^, along with those of the control group, revealed an intact capsule and preserved parenchyma with centrilobular veins, sinusoidal capillary networks, and hepatocytes exhibiting well-defined nuclei and membranes (Figure 4). However, small inflammatory infiltrates were identified in the liver sections of animals treated with AgNPs-Cb, Cb, and Ag^+^ (Figure 4C,F,I). For instance, Su et al. evaluated the toxicity and dissolution rate of AgNPs in rats in vivo, 24 h after intravenous administration at 500 μg/kg, reporting a quantitatively higher concentration of Ag^+^ in the spleen, kidneys, lungs, and brain, with comparatively lower levels in the liver [35]. Their histological analysis showed inflammatory infiltrates and necrotic areas in the liver, with no morphological changes in the kidneys, lungs, or brain. Unlike Su et al., who used higher doses (500 μg/kg), our low-dose AgNPs-Cb (64 μM) showed no lung toxicity, underscoring dose-dependent effects. Therefore, the inflammatory infiltrates seen in the livers of animals treated with AgNPs-Cb in this study could potentially be attributed to the presence of Ag^+^ ions, resulting from AgNPs-Cb metabolism and dissolution within the liver.

The mechanisms of action of AgNPs in biological systems are multifaceted [36]. A study demonstrated that intravenous injection of AgNPs at a single dose induced inflammatory infiltrate formation in the liver within 4 h of exposure, peaking at 72 h and decreasing by the seventh day [23]. Interestingly, this same study noted that the intravenous administration of AgNO_3_ failed to induce an inflammatory process, suggesting distinct toxicity mechanisms between free and nanostructured silver [23]. Another study demonstrated that 30 nm AgNPs coated with polyethylene glycol (PEG) and administered intravenously (8 mg/kg) did not cause visible morphological changes in the liver or kidneys of mice after 24 and 48 h of exposure [37].

Furthermore, research on the toxicological properties of extracts from pequi (*C. brasiliense*), especially the leaves, remains limited [38,39]. However, a recent study investigated the toxicity of aqueous extracts from the bark and leaves of *C. brasiliense* [39]. High concentrations of bark extracts (500, 250, and 125 mg/kg) and leaf extracts (300 and 150 mg/kg) injected into mice resulted in complete (100%) mortality within the first 24 h following intraperitoneal administration [39]. Therefore, the inflammatory infiltrates observed in the liver sections of animals exposed to the aqueous extract of pequi leaves (Cb) may be associated with the presence of bioactive compounds in the extract, even at much lower concentrations.

**Figure 5:**
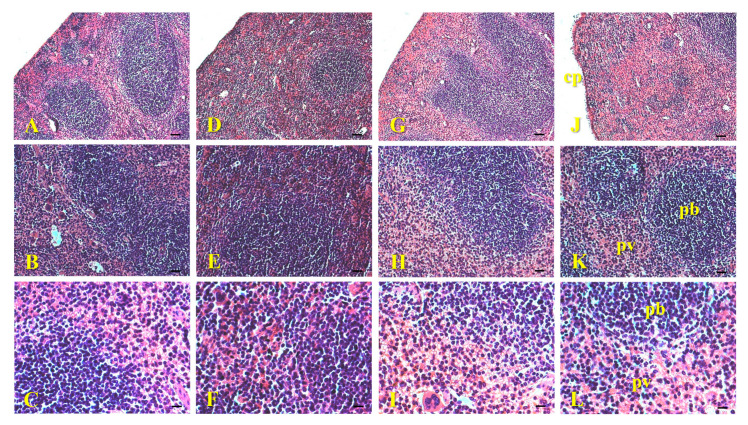
Histological images of mice spleen (C57Bl/6) 24 h after intravenous administration of 64 μM of AgNPs-Cb (**A**–**C**), Cb (**D**–**F**), AgNO_3_ (**G**–**I**), and the untreated control group (**J**–**L**). The sections show whole capsules (cp) (**A**,**D**,**G**,**J**), parenchymal regions divided into white pulp (pb) and red pulp (pv) (**B**,**C**,**E**,**F**,**H**,**I**,**K**,**L**) with no indication of morphological changes. Staining: H&E. Scale bars = 50 μm (**A**,**D**,**G**,**J**); 25 μm (**B**,**E**,**H**,**K**); and 12.5 μm (**C**,**F**,**I**,**L**). Section thickness = 5 μm.

The histological sections of the spleen of animals exposed to AgNPs-Cb, Cb, AgNO_3_, and the control showed intact capsules and well-preserved parenchyma, with the white pulp consisting of lymph nodes and red pulp rich in red blood cells (Figure 5). No morphological changes, inflammatory infiltrates near blood vessels, or clusters of nanoparticles were observed (Figure 5). In a recent study, AgNPs were injected intravenously (7.5, 30, and 120 mg/mL), and after 7 and 14 days, the mice were euthanized [30]. No notable histopathological differences were observed in the brain, heart, spleen, kidneys, and ovaries in any experimental animals. Additionally, molecular analyses of the organs revealed a higher presence of silver in the liver and spleen, followed by the testes, kidneys, brain, and lungs. However, it was observed that the silver concentration in the control group (AgNO_3_) was higher compared with the groups that received AgNPs [30]. In addition to this study, spherical AgNPs with 20, 80, and 100 nm were administered intravenously for 28 days in rats [40]. In that study, the authors demonstrated, by molecular analysis that the AgNPs with 20 nm were distributed mainly in the liver, followed by the kidneys and spleen. In comparison, the larger particles with 80 and 100 nm were distributed mainly in the spleen, followed by the liver and lungs [40], indicating size-dependent tissue targeting.

**Figure 6:**
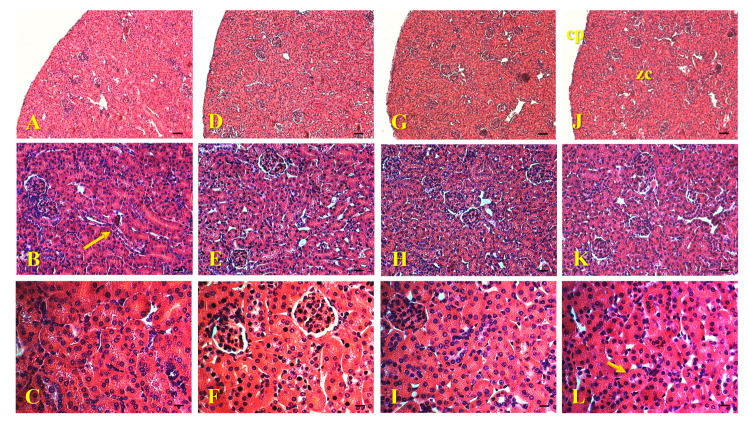
Histological images of mice kidneys (C57Bl/6) 24 h after intravenous administration of 64 μM AgNPs-Cb (**A**–**C**), Cb (**D**–**F**), AgNO_3_ (**G**–**I**), and the untreated control group (**J**–**L**). The sections show intact capsules (cp) and preserved cortical zones (zc) (**A**,**D**,**G**,**J**). In the cortical zone, it is possible to observe renal corpuscles, distal convoluted tubules (large arrow), and proximal convoluted tubules (small arrow) with preserved characteristics (**B**,**C**,**E**,**F**,**H**,**I**,**K**,**L**) with no indication of morphological changes. Staining: H&E. Scale bars = 50 μm (**A**,**D**,**G**,**J**); 25 μm (**B**,**E**,**H**,**K**); and 12.5 μm (**C**,**F**,**I**,**L**). Section thickness = 5 μm.

The histological sections of the kidneys of animals exposed to AgNPs-Cb, Cb, Ag^+^, and the control showed intact capsules and preserved cortical zones (Figure 6). It was possible to observe renal corpuscles, distal convoluted tubules, and proximal convoluted tubules with normal and preserved characteristics (Figure 6), with no indication of morphological changes or AgNPs clusters. As previously reported, Lankveld et al. evaluated the effect of AgNPs (spherical with 20, 80, and 100 nm) administered in single and multiple doses for 28 days in mice [40]. The authors observed a time-dependent effect in which a single dose of AgNPs caused morphological changes and signs of inflammation. In multiple doses, they induced minor peripheral inflammation and damage to the glomerular membrane in the kidneys [40]. Additionally, no significant differences related to particle size were identified.

**Figure 7:**
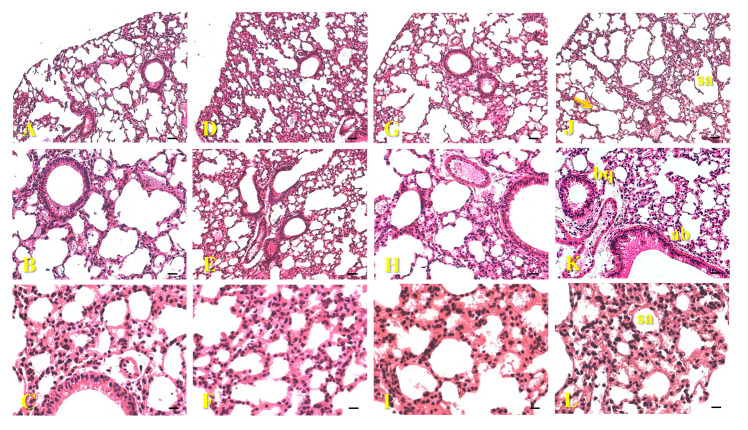
Histological images of mice lungs (C57Bl/6) 24 h after intravenous administration of 64 μM of AgNPs-Cb (**A**–**C**), Cb (**D**–**F**), AgNO_3_ (**G**–**I**), and the untreated control group (**J**–**L**). In the sections, alveolar sacs (sa), bronchioles (bq), and capillaries (arrow) were observed with preserved characteristics for all groups (**B**,**C**,**E**,**F**,**H**,**I**,**K**–**L**). Staining: H&E. Scale bars = 50 μm (**A**,**D**,**G**,**J**); 25 μm (**B**,**E**,**H**,**K**); and 12.5 μm (**C**,**F**,**I**,**L**). Section thickness = 5 μm.

The histological sections of the lungs of animals exposed to AgNPs-Cb, Cb, AgNO_3_, and the control showed preserved characteristics. Alveolar sacs, bronchioles, alveoli, bronchial arteries, and capillaries in all groups showed preserved characteristics, with no indication of morphological changes or AgNP clusters (Figure 7).

Morphological changes in lung tissues after exposure to AgNPs have been reported in the literature [23,30,41,42]. Xue and collaborators (2012) demonstrated that higher concentrations of AgNPs led to the development of small interstitial edemas and the appearance of inflammatory infiltrates (30 mg Kg^−1^ after 14 days) and an increase in the thickness of the alveolar walls, in addition to foci of inflammatory infiltrates (120 mg kg^1^ after 7 days) [30]. Additionally, intravenous administration of AgNPs (25 μg of Ag^+^) in mice led to fibrous hyperplasia, observed in the vicinity of blood vessels in the lung after 24 h of exposure [40]. These findings are in contrast to those found in the present study, which may be related to differences in the performance of the tests, such as dose, exposure time, and synthesis route used to obtain AgNPs.

In vivo studies in rodents (mice and rats) have been developed using different exposure routes to characterize the tissue distribution kinetics and potential toxic effects of AgNPs [30,43,44,45,46,47]. The results related to adverse effects after the administration of AgNPs have still been quite controversial. Some studies indicate that AgNPs may have toxic effects on the liver, kidneys, lungs, small intestine, nervous system, and immune system [28,30,43,47,48]. However, other studies report that AgNPs do not cause relevant adverse effects [41,45,46,47]. These contradictory findings may be related to the variability in the characteristics of the AgNPs investigated, such as the method of production (synthesized in the laboratory or commercially acquired), synthesis routes used (chemical, physical, or biological), size, shape, surface charge, dispersion state, coating agent, and concentration evaluated. In addition, variations related to the characteristics of the animals, such as sex, age, and experimental design (dose, exposure time, and number of animals), may also influence the results obtained and contribute to these divergent effects reported by several authors [41].

Overall, the AgNPs-Cb from the present study showed no evidence of relevant toxicity either in vitro or in vivo, even in aspects that contrast with the literature for similar concentrations. However, unlike most previous studies, which utilized AgNPs synthesized through chemical routes, this work employed a distinct approach using AgNPs synthesized via a green route. This difference may be decisive in explaining the results obtained, suggesting that the green synthesis route imparts unique characteristics to the nanoparticles, such as greater biocompatibility and stability. These findings are highly relevant to the literature, as they not only challenge previous interpretations but also open new possibilities for the development of safer and more sustainable silver-based materials. Future studies can further explore the impact of synthesis routes on the biological properties of AgNPs, expanding their applicability across various fields, from nanomedicine to agroindustry, with a focus on safety and efficacy

## 3. Materials and Methods

### 3.1. Materials

Analytical grade reagents and ultrapure water were used for the solutions’ preparation. The chemicals included colorless acrylic varnish (Acrilex, São Bernardo do Campo, Brazil), ethylenediamine tetraacetic acid (EDTA) (Proquímios, Rio de Janeiro, Brazil), hematoxylin (Dinâmica, Indaiatuba, Brazil), ethanol (Dinâmica, Brasil), eosin (Dinâmica, Brazil), Methyl-Carnoy fixative (methanol PA 60% (Proquímios, Brazil), chloroform 30% (CRQ, São Paulo, Brazil), and acetic acid 10% (Merck, Rio de Janeiro, Brazil)), phosphate-buffered saline (PBS) (Laborclin, Pinhais, Brazil), ketamine (Syntec, Barueri, Brazil), nonionic surfactant Triton X-100 (Sigma-Aldrich Co., St. Louis, MO, USA), Paraplast (Sigma-Aldrich Co., USA), silver nitrate (AgNO_3_) (Plat-Lab, Guarulhos, Brazil), xylazine hydrochloride (Syntec, Brazil), and xylene (Sigma-Aldrich Co., USA).

### 3.2. Synthesis and Characterization of Silver Nanoparticles

The leaf extract of *C. brasiliense* Camb was used for green synthesis of AgNPs, with authorization for access to genetic patrimony (CGEN n. 02001.007580/2014-95). Frozen leaves were washed with diluted detergent, rinsed, air-dried, and cut into fragments (~5 mm). Fragments were boiled in ultrapure water (100 mg/mL) for 2 min. The extract was gravity-filtered (Whatman No. 1) under light protection. One aliquot was used immediately for the AgNPs’ synthesis. AgNPs-Cb were synthesized following the protocol previously described by Bonatto in 2016 [15], as follows: 500 µL of leaf extract (Cb extract) was added to 49.5 mL of 1 mM aqueous AgNO_3_ solution (Plat-Lab, Guarulhos, Brazil) under dark conditions at 75 °C for 150 min. Afterward, 25 mL of colloidal suspension was stored in a 50 mL polypropylene tube and frozen at −80 °C for 4 h. The sample was then lyophilized and reconstituted by adding 2.5 mL of ultrapure water. Characterization was performed using an absorption curve at 350–550 nm to assess the maximum absorption peak and by dynamic light scattering (DLS).

### 3.3. Dynamic Light Scattering (DLS) and Zeta Potential

The hydrodynamic diameter and polydispersity index (PdI) of the AgNPs were analyzed by photon correlation spectroscopy, and the zeta potential was analyzed by electrophoretic mobility using a ZetaSizer Nano ZS (Malvern Instruments, Worcestershire, UK) with a 4 mW He-Ne laser at 633 nm, detecting light at 173°. Three measurements were taken in the automatic mode at room temperature, and data were processed using ZetaSizer version 7.11 software.

### 3.4. Atomic Force Microscopy

One μL of AgNPs-Cb suspension was deposited on freshly cleaved muscovite mica and air-dried in a protected environment. The sample was mounted on a metal holder with double-sided adhesive tape. The analysis was conducted in ambient air at 22 °C with a commercial atomic force microscope (Shimadzu SPM-9600, Kyoto, Japan) equipped with a scanner with a maximum scan area of 125 μm × 125 μm. The dynamic phase mode was applied using a rectangular cantilever with a conical silicon tip (spring constant of 10–130 N/m and resonance frequency range of 204–497 kHz), and a sweep acquisition rate of 1 Hz. Images were captured of 10 μm × 10 μm areas at a 512 × 512 lines resolution and processed for plane correction and segmentation with the instrument’s offline software (version 3.304).

### 3.5. Transmission Electron Microscopy

The AgNPs-Cb were also analyzed for morphology by TEM. For sample preparation, 5 μL of AgNPs-Cb suspension, 10-fold diluted, were deposited onto 150-mesh copper grids coated with a carbon-coated Formvar film. The material was then kept under protective conditions in a dry environment at 25 °C for at least 24 h for complete drying. The morphology of the synthesized AgNPs was analyzed using a JEM-1011 (Jeol, Tokyo, Japan) transmission electron microscope operated at 100 kV. Electron micrographs were acquired with a digital camera (Gatan, Pleasanton, CA, USA) integrated with the microscope. The AgNPs-Cb length (diameter) was measured using ImageJ software (version 1.54 g).

### 3.6. Hemolysis in Vitro

Blood from C57Bl/6 mice was collected from the orbital sinus into polypropylene microtubes containing 10 μL of 10% EDTA and stored at 4°C. Seventy μL of whole blood were incubated with 10 μL of the AgNPs-Cb suspension or control solutions (Cb extract and free Ag^+^ (AgNO_3_) at concentrations of 128, 64, 32, 16, and 8 μM) at 37 °C with gentle agitation for 30 min. Triton X-100 (0.2%) served as the positive control, and PBS (100 mM) as the negative control. The assay was performed in triplicate. The samples were centrifuged at 900× *g* for 5 min, and 1 μL of the supernatant was transferred to a 96-well polystyrene microplate with 199 μL of ultrapure water. The absorbance was measured at 405 nm, and hemolysis was calculated relative to the positive control (100% hemolysis).

### 3.7. Toxicity in Mice

Adult C57Bl/6 mice (~8 weeks old, healthy without visible anomalies) were housed under a 12-hour light/dark cycle at 23 °C and 55% humidity with ad libitum access to food and water in the Animal Facility of the Institute of Biological Sciences, University of Brasília. Mice were randomly divided into four groups (five/six animals per group) and weighed before receiving a single intravenous injection (tail vein) of 20 μL of AgNPs-Cb (n = 6), Cb extract (n = 5), or free Ag^+^ (AgNO_3_-solution, n = 5) at approximately 64 μM of AgNPs-Cb equivalent in silver, considering 2.5 mL of blood volume per animal. The control group (n = 5) did not receive any treatment. The experiments prioritized an ethical reduction in animal use while maintaining statistical robustness for hematological and biochemical endpoints. All animals met predefined health/age criteria and were included in the analysis without post-allocation exclusions. Treatments were administered in randomized order, and animals were housed in uniform environmental conditions (temperature and light cycles) to minimize location- or sequence-related confounders. Group allocation was known only to the in vivo experimenters during treatment administration. Outcome assessments (hematological and biochemical) were conducted by blinded analysts using automated systems. Mice were reweighted 24 h post-injection, anesthetized with ketamine and xylazine, and euthanized for organ collection (blood, spleen, liver, kidneys, and lungs) for hematological, biochemical, and histological analyses. Ethical approval was obtained from the Institute of Biological Sciences Ethics Committee, University of Brasília (no. 131758/2012).

### 3.8. Hematological and Biochemical Evaluations

Peripheral blood from mice was collected through the orbital sinus, and 200 μL was stored in ethylenediaminetetraacetic acid (EDTA)-containing microtubes at 4 °C. Blood samples were analyzed for the complete blood count and leukogram. Serum was isolated from blood without anticoagulant for biochemical analysis, including total bilirubin and fractions, gamma glutamyl transferase (GGT), aspartate aminotransferase (AST), alanine aminotransferase (ALT), glucose, and lipid profile (Table 5). Tests were conducted in collaboration with the Sabin Institute through the Research Support Center.

### 3.9. Histological Evaluation

Liver, spleen, kidney, and lung fragments were fixed with Methacarn fixative (Methyl-Carnoy) for 6 h at 22 °C. Samples were hydrated in decreasing concentrations of ethyl alcohol (90%, 80%, and 70%) and preserved in 70% ethyl alcohol for 48 h at room temperature. The samples were then dehydrated in increasing concentrations of ethyl alcohol (80%, 90%, and 100% (twice)), cleared in xylene for 40 min (ethanol/xylene 1:1, xylene 1, xylene 2, and xylene 3), and embedded in Paraplast^®^ through two 1.5-hour immersions (Paraplast^®^ 1 and Paraplast^®^ 2). After embedding, the samples were molded into Paraplast^®^ blocks using plastic molds. Once solidified, the samples were removed from the molds, stored at 4 °C until microtomy, and then sectioned on a microtome into 5 μm thick slices. The slices were placed on glass slides and incubated at 37 °C for 48 h. Subsequently, hematoxylin and eosin (H&E) staining was performed. The slides underwent removal of the embedding medium (Paraplast^®^) with successive xylene baths for 3 min (xylene 1, xylene 2, and xylene/ethanol 1:1), hydrated in decreasing concentrations of ethyl alcohol for 2 min each (100%, 90%, 80%, and 70%), and washed in distilled water for 2 min. Slides were then immersed in hematoxylin stain for 1 min, rinsed with running water, and stained with eosin for 30 s. The material was dehydrated in increasing concentrations of ethyl alcohol (70%, 80%, 90%, and 100%) and xylene (xylene 1 and xylene 2) for 20 s each. After the final xylene bath, the slides were covered with coverslips using colorless acrylic varnish as the mounting medium and left to dry in an oven at 37 °C for 12 h. Histological slides were analyzed under an Axiophot light microscope (Zeiss, Oberkochen, Germany) equipped with a digital camera (Zeiss) and digitally documented using AxioVision software version 4.8, which was available at the Bioimaging Laboratory of Embrapa Genetic Resources and Biotechnology.

### 3.10. Statistical Analysis

Data are expressed as the mean ± standard error of the mean (SEM). Statistical analysis was performed with PAST software (version 2.17b) [49], with possible significant differences among groups evaluated using analysis of variance (ANOVA) and Tukey’s post hoc test, with significance set at *p* < 0.05.

## 4. Conclusions

This study demonstrates that AgNPs synthesized via a green route using the aqueous extract of *C. brasiliense* leaves (AgNPs-Cb) exhibited very-low acute toxicity in a murine model. Hematological and biochemical analyses revealed no significant alterations, while histological evaluations indicated only small inflammatory infiltrates in the liver, likely caused by residual bioactive compounds in the aqueous extract. Additionally, AgNPs-Cb exhibited a very-low hemolytic rate, preserving over 98% of red blood cells even at the highest concentration tested. These findings underscore the potential of green-synthesized AgNPs-Cb as an alternative for antimicrobial applications compared to conventional chemically synthesized AgNPs or free silver ions. The use of green synthesis not only aligns with environmentally friendly principles but also appears to enhance the biocompatibility of the AgNPs. This unique synthesis approach, which contrasts with the chemical routes used in most studies, highlights the importance of exploring sustainable methods to improve nanoparticle safety profiles. However, further studies are essential to elucidate the long-term effects, biodistribution, mechanisms of action, and interactions of AgNPs-Cb in complex biological systems. Such investigations will not only validate the current findings but also pave the way for expanding their potential applications in nanomedicine and other fields.

## Figures and Tables

**Table 1:** Analysis of hematological parameters (red series) of mice (C57Bl/6) 24 h after intravenous administration of AgNPs-Cb, Cb, and AgNO_3_. Values are presented as the mean ± standard error of the mean, n = 5.

	Red Blood Cells (million/mm^3^)	Hemoglobin (g/dL)	Hematocrit (%)	MCV (fl)	MCH (pg)	MCHC (g/dL)	RDW (%)
AgNPs-Cb	9.14 ± 0.15	13.40 ± 0.32	43.72 ± 0.64	47.87 ± 0.47	15.02 ± 0.12	30.98 ± 0.39	18.35 ±0.64
Cb	8.67 ± 0.40	12.59 ± 0.88	42.35 ± 2.22	48.75 ± 0.41	14.85 ± 0.36	31.28 ± 0.17	17.28 ± 0.98
AgNO_3_	9.21 ± 0.19 *	14.00 ± 0.21	45.00 ± 0.49 *	48.78 ± 0.73	15.18 ± 0.18	31.10 ± 0.32	16.80 ± 0.68
Control	8.27 ± 0.32	12.40 ± 0.64	40.00 ± 1.77	48.10 ± 0.59	14.90 ± 0.29	31.40 ± 0.21	15.73 ± 1.14
Reference ^#^	7.14–12.20	10.8–19.2	37.3–62.0	42.7–56.0	11.7–16.8	24.6–35.9	15.9–21.1

* Statistically significant difference (*p* < 0.05) compared with the control. MCV = mean corpuscular volume; MCH = mean corpuscular hemoglobin; MCHC = mean corpuscular hemoglobin concentration; RDW (red cell distribution width) = distribution of the width of red blood cells. ^#^ Charles River research models: C57Bl/6 mouse hematology.

**Table 2:** Analysis of hematological parameters (white series) of mice (C57Bl/6) 24 h after intravenous administration of AgNPs, Cb, and AgNO_3_. Values are presented as the mean ± standard error of the mean, n = 5.

	Leukocytes (%mm^3^)	Lymphocytes (%/mm^3^)	Segmented (%/mm^3^)	Eosinophils (%/mm^3^)	Basophils (%/mm^3^)	Monocytes (%/mm^3^)
AgNPs-Cb	6883.33 ± 457.83	82.00 ± 3.02	16.00 ± 2.21	0.33 ± 0.33	0.00 ± 0.00	1.50 ± 0.61
Cb	6500.00 ± 1361.98	88.50 ± 1.94	10.75 ± 1.55	0.00 ± 0.00	0.00 ± 0.00	0.75 ± 0.38
AgNO_3_	6480.00 ± 863.94	86.00 ± 2.98	12.80 ± 2.56	0.00 ± 0.00	0.00 ± 0.00	1.20 ± 0.54
Control	8725.00 ± 1652.96	84.00 ± 4.36	15.25 ± 3.97	0.00 ± 0.00	0.00 ± 0.00	0.75 ± 0.48
Reference ^#^	-	61.26–87.82	7.36–28.59	0.13–4.51	0.00–1.26	2.18–11.02

There was no statistically significant difference (*p* < 0.05) compared with the control. ^#^ Charles River research model: C57Bl/6 mouse hematology.

**Table 3:** Hepatic function biomarkers and bilirubin fractions in the blood serum of mice (C57Bl/6) 24 h after intravenous administration of AgNPs-Cb, Cb, and AgNO_3_. Values are presented as the mean ± standard error of the mean, n = 5.

	AST (U/L)	ALT (U/L)	GGT (U/L)	TBIL (mg/dL)	DBIL (mg/dL)	IBIL (mg/dL)
AgNPs-Cb	122.33 ± 1.87	62.50 ± 9.44	12.00 ± 3.22	0.77 ± 0.07	0.37 ± 0.05	0.40 ± 0.05
Cb	101.80 ± 12.60	47.60 ± 7.30	4.80 ± 1.02	0.65 ± 0.12	0.25 ± 0.08	0.39 ± 0.08
AgNO_3_	108.00 ± 6.44	50.40 ± 6.18	14.60 ± 5.37	0.78 ± 0.12	0.26 ± 0.09	0.52 ± 0.08
Control	137.00 ± 0.63	50.00 ± 7.16	7.80 ± 3.15	0.62 ± 0.17	0.24 ± 0.11	0.39 ± 0.13
Reference ^#^	43–397	27–195	0–9	0.2–0.6	-	-

There was no statistically significant difference (*p* < 0.05) compared with the control. Gamma glutamyl transpeptidase (GGT), aspartate aminotransferase (AST), alanine aminotransferase (ALT), total bilirubin (TBIL), direct bilirubin (DBIL), and indirect bilirubin (IBIL). ^#^ Charles River research models: C57Bl/6 mouse hematology.

**Table 4:** Blood glucose and lipid profile parameters in the blood serum of mice (C57Bl/6) 24 h after intravenous administration of AgNPs-Cb, Cb, and AgNO_3_. Values are presented as the mean ± standard error of the mean, n = 5.

	Glucose (mg/dL)	Total Cholesterol (mg/dL)	HDLs (mg/dL)	LDLs (mg/dL)	Non-HDLs (mg/dL)	Triglycerides (mg/dL)
AgNPs-Cb	66.83 ± 10.18	137.33 ± 20.10	39.83 ± 5.88	62.67 ± 18.74	97.50 ± 21.88	173.33 ± 32.19
Cb	81.80 ± 26.87	112.80 ± 18.58	44.8 ± 5.34	44.80 ± 20.14	68.00 ± 22.82	116.60 ± 20.93
AgNO_3_	92.80 ± 25.66	128.00 ± 19.00	45.00 ± 4.94	59.80 ± 17.16	83.00 ± 21.60	117.00 ± 20.22
Control	91.25 ± 20.63	95.60 ± 4.37	48.60 ± 8.54	26.80 ± 9.01	47.00 ± 9.50	104.20 ± 10.46
Reference ^#^	43–397	77.24–209.5	-	0.2–0.6	-	98.16–209.58

There was no statistically significant difference (*p* < 0.05) compared with the control. Low-density lipoproteins (LDLs) and high-density lipoproteins (HDLs). ^#^ Charles River research models: C57Bl/6 mouse hematology.

**Table 5:** Methodologies used by the Sabin Clinical Analysis Laboratory for hematological and biochemical evaluations of blood samples from animals.

Parameter	Methodology
Hemogram	Fluorescent flow cytometry and impedance
Leukogram	Fluorescent flow cytometry and impedance
Total bilirubin and fractions	Oxidation with vanadate
Gamma glutamyltransferase (GGT)	Kinetic colorimetric
Aspartate aminotransferase (AST)	Kinetic optimized UV
Alanine aminotransferase (ALT)	Kinetic optimized UV
Glucose	Enzymatic colorimetric
Lipidogram	Enzymatic colorimetric

## Data Availability

The original contributions presented in the study are included in the article, further inquiries can be directed to the corresponding authors.

## Abbreviations

The following abbreviations are used in this manuscript:

ALT	Alanine aminotransferase
AFM	Atomic force microscopy
AST	Aspartate aminotransferase
AgNPs	Silver nanoparticles
Cb	Aqueous extract of *Caryocar brasiliense* leaves
CBC	Complete blood count
DBIL	Direct bilirubin
DLS	Dynamic light scattering
GGT	Gamma glutamyl transpeptidase
HDLs	High-density lipoproteins
IBIL	Indirect bilirubin
LDLs	Low-density lipoproteins
MCH	Mean corpuscular hemoglobin
MCHC	Mean corpuscular hemoglobin concentration
MCV	Mean corpuscular volume
MNPs	Metal nanoparticles
PdI	Polydispersity index
RBCs	Red blood cells
RDW	Red cell distribution width
RPS	Refractive plasmonic scattering
TBIL	Total bilirubin
TEM	Transmission electron microscopy
